# Unveiling the Puzzle: Reactivated Epstein-Barr Virus-Associated Pancreatitis in a Young Adult

**DOI:** 10.7759/cureus.79643

**Published:** 2025-02-25

**Authors:** Michael K Coffin, Michael Zatoulovski, Zulfiya Tashpulatova, Jacob De Castro, Steven Johnson

**Affiliations:** 1 Family Medicine, William Carey University College of Osteopathic Medicine, Hattiesburg, USA; 2 Gastroenterology, William Carey University College of Osteopathic Medicine, Hattiesburg, USA

**Keywords:** ebv (epstein-barr virus), ebv pathogenesis, serum lipase, severe acute pancreatitis, treatment of pancreatitis

## Abstract

Epstein-Barr virus (EBV) is a rare cause of acute pancreatitis, with only 17 cases reported in the literature. We describe a 25-year-old male patient with recurrent pancreatitis, ultimately attributed to EBV reactivation. The initial presentation included severe epigastric pain, elevated lipase levels, and unremarkable imaging. A subsequent episode revealed diffuse pancreatic inflammation on MRI and positive Epstein-Barr nuclear antigen, confirming the diagnosis. Other common etiologies, including alcohol use, gallstones, and genetic predisposition, were ruled out. The patient was successfully managed with supportive care. This case highlights the diagnostic challenges of EBV-related pancreatitis and underscores the importance of considering viral etiologies in recurrent pancreatitis cases.

## Introduction

Pancreatitis is an inflammation leading to the auto-digestion of this vital organ by its digestive enzymes. While the cardinal signs of pancreatitis are well known, the challenge comes in delaying the progression of this acute process as it can result in multi-system organ failure and death in about 20% of cases [1]. It can be as simple as providing conservative care as the pancreatitis resolves by itself to more invasive measures depending on the cause. While viral etiologies are rare, the Epstein-Barr virus (EBV) has been identified as a potential trigger of pancreatitis in 17 cases reported in the literature [2-4]. Approximately 10% of EBV infections are asymptomatic; however, in rare instances, the virus can lead to pancreatitis [5]. This form of pancreatitis is typically self-limiting, driven by an inflammatory reaction to the virus, and often resolves as the immune system clears the infection [6]. According to the literature, this is usually a one-time occurrence in children and young adults with a primary mononucleosis infection and does not occur again as any recurrences are much milder [6]. The reactivation of the EBV is a well-documented occurrence and has been linked to immune suppression and other pathogens. When it is reactivated, in either of these cases or in the immunocompetent, symptoms can vary from lack of symptoms to fatigue and upper respiratory symptoms. It can cause elevated aminotransferases and splenomegaly as well [7]. Pancreatitis resulting from EBV infection is an infrequent occurrence. We present this case to contribute to the limited body of evidence and to explore the management of this uncommon etiology.

## Case presentation

History of presentation 

We present a 25-year-old male patient, with a past medical history (PMHx) of myocarditis secondary to mycoplasma pneumonia, who presented to the emergency department for severe epigastric pain, radiating to the back, and escalating over several days. The patient denied alcohol use of any kind. His medical history was negative for the risk of heart disease after his recovery from myocarditis. The patient denied symptoms of crushing chest pain, perfuse sweating, or radiating pain to either shoulder that could point to an MI. The patient denied epigastric pain related to the ingestion of food that could be related to peptic ulcer disease. He also denied having right upper quadrant (RUQ) pain, acholic stools, fever, or noticeable jaundice that could relate to hepatitis. Physical exam was remarkable for a very tender epigastric region with reproducible pain on palpation and rebound tenderness but without tenderness to any specific quadrant of the abdomen. Heart sounds were unremarkable except for some moderate tachycardia of 120 bpm (reference range: 60-100 bpm). Lower diffuse back pain was present from the thoracic vertebrae T5 to T9 but was not reproducible by palpation. Bowel sounds were normoactive x4. The sclera were anicteric. 

Initial laboratory values revealed a white blood cell (WBC) count of 15,000/μL (reference range: 4,500-11,000/μL)and an elevated lipase level of >1000 units per liter or U/L (reference range: 0-160 U/L). All other labs, including triglycerides, bile acids, blood urea nitrogen (BUN), amylase, and aspartate transaminase (AST)/alanine transaminase (ALT) were normal. Given his work history as a bartender, it was presumed that he wasn't being forthcoming about his alcohol consumption. He was presumptuously diagnosed with alcoholic pancreatitis. He received symptomatic management with morphine and IV fluids, and computed tomography (CT) of the abdomen/pelvis (A/P) which showed no abnormalities. The patient also underwent a magnetic resonance imaging (MRI) of the lumbar spine to rule out musculoskeletal pathologies, but imaging of the pancreas was not within view. The impression stated that there were no bulging discs, degenerative disorders, or any other musculoskeletal reason for his back pain. His ethyl alcohol (EtOH) results came back shortly after and were within normal limits. It was concluded that his pancreatitis had been caused by a different etiologic agent.

**Figure 1:**
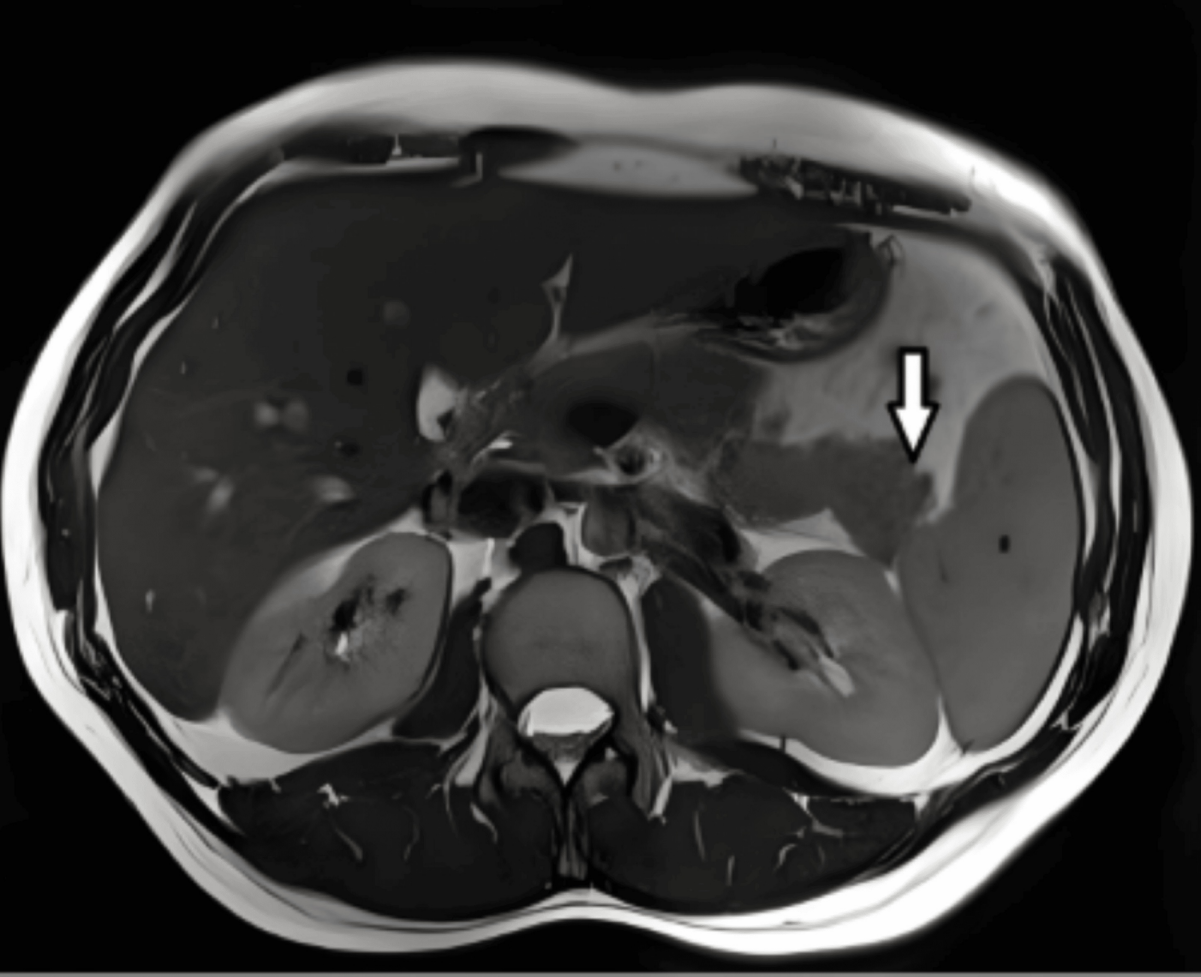
Axial T1-weighted MRI demonstrates decreased signal intensity in the pancreas, consistent with acute inflammation and edema. The pancreas appears mildly enlarged with irregular contours. The arrow indicates the inflamed pancreas.

One month later, the patient returned to the emergency department with worsening and similarly presenting sudden-onset abdominal pain. He characterized the pain as a 10/10, and it had started abruptly after eating a fatty meal. He stated that it was achy in nature, and only improved slightly with leaning forward and was exacerbated when lying flat. The physical examination was identical to his previous visit. His labs showed elevated lipase again. An MRI at the level of the entire thoracic spine was obtained to view the pancreas and the abdominal viscera as a whole. This revealed diffuse inflammation in the tail of the pancreas (Figure 1).

Viral panel results were predominantly negative, except for the presence of Epstein-Barr nuclear antigen. The patient then underwent an upper endoscopy that revealed no gallstones or signs of ongoing gastric inflammation. Genetic testing for pancreatic secretory trypsin inhibitor (SPINK1), cystic fibrosis transmembrane conductance regulator (CFTR), cationic trypsinogen (PRSS1), and chymotrypsinogen (CTRC) were negative. He was admitted to the unit for three days. During this time, besides the elevated WBC count and tachycardia due to pain, the patient had no signs that indicated he was going through a systemic inflammatory response syndrome (SIRS). His temperature was 37.2°C and the respiratory rate was 18 breaths per minute. The CT scan reflected a Balthazar score of zero which indicated a grade A pancreas. Therefore, without anything acute to tackle, the patient was discharged home with hydrocodone for pain management. He was referred to his primary care physician (PCP) for routine follow-up, outpatient GI for a potential upper endoscopy, and an immunologist for his reactivated EBV.

Investigations

When the patient first presented with the classic symptoms of upper epigastric pain that radiates to the back, an immediate lipid panel and lipase level was ordered and showed unremarkable low-density lipoprotein (LDL), high-density lipoprotein (HDL), and triglyceride levels, and a lipase level of >1500 U/L (Table 1). A subsequent CT scan with and without contrast was ordered and showed no acute inflammation of the pancreas so the patient was stabilized and discharged.

**Table 1:** Laboratory values throughout the patient's recovery This table presents the patient's laboratory values across two emergency room (ER) admissions and a six-month follow-up, highlighting values that were outside the reference range. Lipase was markedly elevated at the first ER admission (>10,000 U/L), significantly decreased at the second ER visit (2,000 U/L), and normalized at follow-up (84 U/L), consistent with the resolution of the recurrent acute pancreatitis. All other laboratory values remained within the reference range throughout the patient's course. HDL-C: High-density lipoprotein cholesterol; LDL-C: Low-density lipoprotein cholesterol; TG: Triglycerides; Chol: Cholesterol

Test	ER admission 1	ER admission 2	Follow-up after six months	Reference ranges
Albumin (g/dL)	4.9	4.3	5.0	3.5-5.0
Bilirubin total (mg/dL)	0.6	0.5	0.4	0.2-1.2
Bilirubin direct (mg/dL)	0.2	0.2	0.2	0-0.5
Aspartate aminotransferase or AST (IU/L)	16	25	24	5-34
Alkaline phosphatase or ALP (IU/L)	56	54	63	40-150
Alanine aminotransferase or ALT (IU/L)	21	31	50	0-50
Protein total (g/dL)	7.1	6.8	8.0	6.7-8.6
Gamma-glutamyl transpeptidase or GGTP (IU/L)	19	21	25	1-64
Lipase (U/L)	>10,000	2000	84	8-78
Total cholesterol (mg/dL)	172	NA	NA	<200
HDL-C (mg/dL)	74	NA	NA	<40
TGs (mg/dL)	42	NA	NA	<150
LDL-C (mg/dL)	86	NA	NA	<100
Chol/HDL-C ratio	2.3	NA	NA	<5.0
Non-HDL-C (mg/dL)	98	NA	NA	<130

When the patient returned the second time, a repeat lipase level was done and was over 500 U/L. As this was the second occurrence, a more thorough panel of herpesviruses, B1, B9, B12, Bartonella, sweat chloride, SPINK1, erythrocyte sedimentation rate (ESR), C-reactive protein (CRP), and liver function tests (LFTs) were taken. The human leukocyte antigen B27 (HLA B-27) was done at the six-month follow-up. All the tests came back normal except for an elevated CRP, a positive HLA-B27, and an abnormal EBV panel (Table 2).

**Table 2:** An EBV panel showing evidence of reactivation of a previous mononucleosis infection EBV: Epstein-Barr Virus; EBV NA: Epstein-Barr Virus Nuclear Antigen; EBV NA RES: Epstein-Barr Virus Nuclear Antigen Result; EBV VCA IGG: Epstein-Barr Virus Viral Capsid Antigen Immunoglobulin G; EBV IGM: Epstein-Barr Virus Immunoglobulin M; U/mL: Units per milliliter; HLA B-27 antigen: Human leukocyte antigen B27

Test Name	Result	Reference
EBV NA	Positive	Negative
EBV NA RES (U/mL)	>600	<14.9
EBV VCA IGG (U/mL)	>750	<17.9
EBV IGM (U/mL)	40	<36.0
HLA B-27 antigen	Positive	Negative
C-reactive protein (mg/dL)	12	<0.3

**Figure 2:**
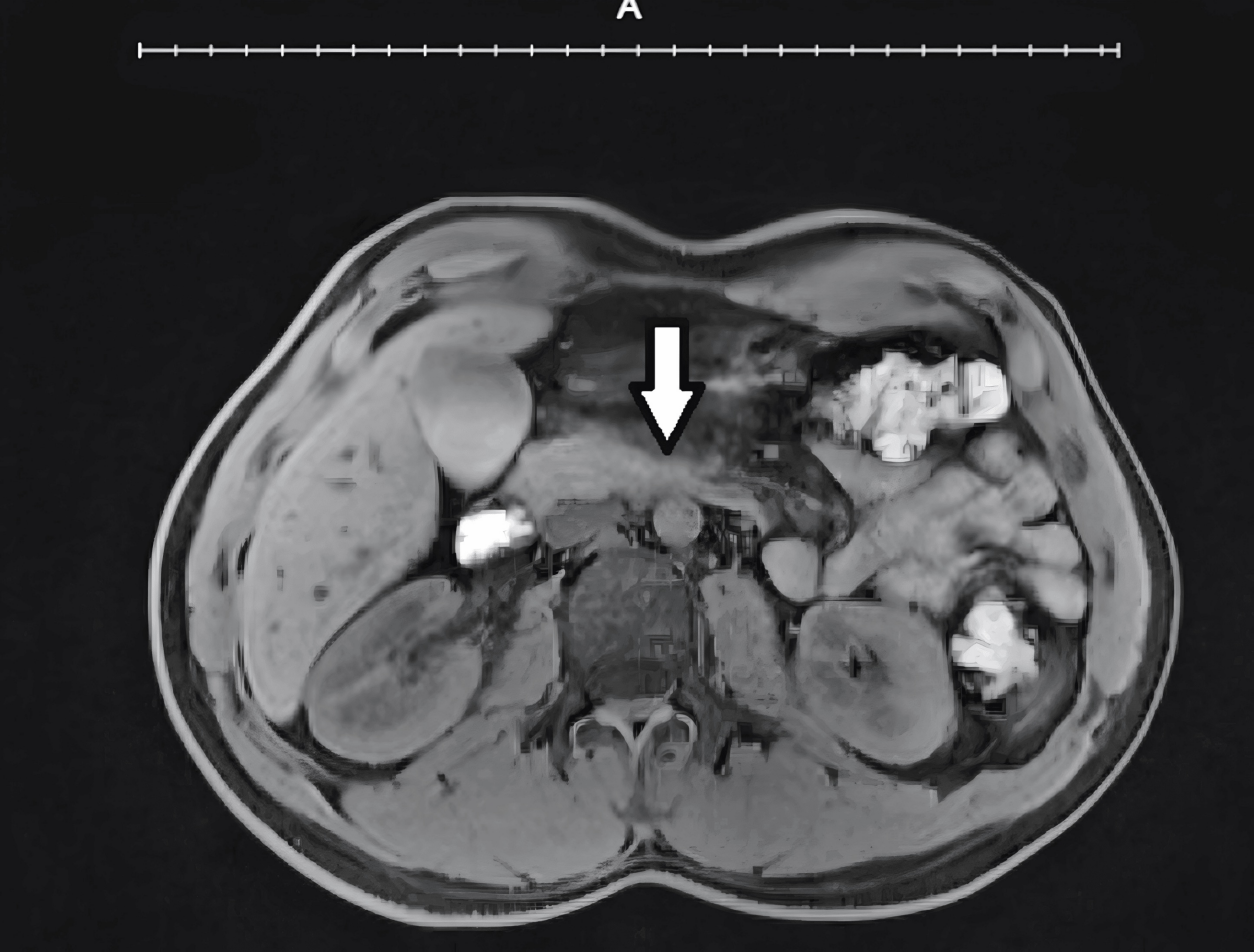
Axial T2-weighted MRI reveals increased signal intensity in the pancreas, indicating inflammatory edema. Hyperintense peripancreatic fluid collections are noted, along with hyperintense inflammatory stranding within the peripancreatic fat, consistent with acute pancreatitis. The arrow indicates the inflamed pancreas.

A repeat CT showed evidence of a recovering inflammation process at the tail end of the pancreas. Once again, the patient was discharged after he was stabilized. He presented one last time three months later for severe back pain which he thought was related to his recurring pancreatitis flares, and underwent an MRI with and without contrast which demonstrated an unremarkable pancreas (Figure 2).

Differential diagnosis 

As soon as the patient presented with epigastric pain that radiated to the back and an enormously high lipase level, we considered his symptoms to be due to pancreatitis secondary to a choledocholithiasis. But this was ruled out with ultrasound. After this, we considered cystic fibrosis as a possible culprit or a viral etiology. The patient’s third visit seemed more musculoskeletal-related rather than GI-related, so we considered musculoskeletal back pain or ankylosing spondylitis due to his positive HLA B-27 result. Duodenal ulcers secondary to Zollinger-Ellison syndrome and malignancy were also considered.

Management 

Upon admission at the third visit, the patient was stabilized with fluid resuscitation and pain management. Diagnostic verifications, including lipase and imaging studies confirmed acute pancreatitis, and virology studies attributed it to the EBV. Antiviral therapy with valacyclovir was initiated, guided by symptomatology, imaging results, lipase, and LFTs. The patient received supportive care, and symptoms were addressed with adequate hydration and pain management. Patient education focused on the viral etiology, causes of acute pancreatitis, and treatment rationale. The discharge plan included follow-up appointments, dietary recommendations, and guidelines for recognizing potential recurrence of symptoms.

Six months after his last emergency room visit, the patient reported feeling significantly better overall, and noted that the experience had left him with more emotional stress than physical sequelae. He mentioned experiencing occasional abdominal discomfort but was able to reassure himself that it was not related to pancreatitis. To address his concerns and promote better gut health, he consulted a nutritionist to develop a balanced dietary plan.

## Discussion

Acute pancreatitis is an inflammatory condition of the pancreas with almost 200,000-250,000 admissions per year [8]. The Revised Atlanta Classification for diagnosing acute pancreatitis includes at least two of the following: abdominal pain consistent with the disease, serum lipase or amylase levels three times above the normal limit, and characteristic findings on CT imaging [9]. The differential diagnosis typically includes common causes of acute pancreatitis such as gallstones, alcohol use, hypertriglyceridemia, medication-induced, and genetic, all of which were excluded based on the patient's history, lab results, and imaging findings. While viral causes of pancreatitis are rare, EBV pancreatitis is particularly uncommon, with only 17 reported cases [2-4].

EBV-associated pancreatitis typically presents alongside infectious mononucleosis in young adults [10]. The diagnosis often requires a high index of suspicion and the exclusion of other more common causes of pancreatitis. EBV, primarily known for causing infectious mononucleosis, can also lead to various other diseases. Common symptoms of EBV infection include fever, sore throat, swollen lymph nodes, and fatigue. The pathophysiological mechanisms by which EBV leads to pancreatitis are not well understood but may involve direct viral invasion of the pancreatic tissue or an immune-mediated response. Recent research has highlighted the potential role of EBV-encoded proteins in modulating immune responses and contributing to the pathophysiology of EBV-associated diseases. Studies have shown the impact of EBV-encoded proteins, such as deoxyuridine triphosphatase (dUTPase), on immune cell functions, including dendritic cells and peripheral blood mononuclear cells [11]. These findings suggest a complex interplay between EBV, its encoded proteins, and the host immune system in the development of EBV-associated diseases.

In most cases, patients with EBV-associated acute pancreatitis present with severe abdominal pain, sore throat, and fever. Laboratory findings frequently reveal elevated pancreatic enzymes such as lipase and amylase, greater than 3x the upper limit [12]. Imaging studies, such as CT scans, usually show inflammation of the pancreas without gallstones or ductal abnormalities. Additionally, patients might have other symptoms of EBV infection, including sore throat, lymphadenopathy, and atypical lymphocytosis. Diagnostic tests for viral pancreatitis include serology for EBV-specific antibodies and polymerase chain reaction (PCR) testing to detect viral DNA. Distinguishing viral etiology from other causes requires a thorough evaluation of the patient's history, lab results, and imaging findings.

In our case, the 25-year-old male patient with a past medical history of myocarditis presented with severe epigastric pain radiating to the back. Notably, the patient lacked typical EBV symptoms such as sore throat and fever. Initial lab results showed elevated WBC count and lipase levels, but other potential causes of pancreatitis were ruled out. Imaging was initially unremarkable, delaying the diagnosis. Upon return to the emergency department with recurrent symptoms, an MRI revealed inflammation in the tail of the pancreas, and serology confirmed EBV infection. 

The patient received symptomatic management with morphine and intravenous fluids during the initial presentation. On readmission, treatment was conservative, including fasting and supportive care until the inflammation subsided. He was discharged with hydrocodone for pain management. This approach aligns with the conservative treatment strategies reported in the literature for EBV-associated pancreatitis and is in line with the American College of Gastroenterology Guidelines for the management of acute pancreatitis [13]. Although antiviral medications such as valacyclovir are not commonly employed for EBV-associated pancreatitis, they have been utilized in two reported cases [14]. Nonetheless, supportive care remains the primary approach for managing this condition.

Recent research on viral pancreatitis, particularly EBV-associated cases, highlights the importance of recognizing this rare etiology and the need for a high index of suspicion. Collaboration with healthcare professionals, including gastroenterologists, infectious disease specialists, and radiologists, is crucial for comprehensive care and optimal patient outcomes.

## Conclusions

This case illustrates the diagnostic challenges of EBV-associated acute pancreatitis, a rare and often overlooked condition. While EBV pancreatitis is typically self-limiting and driven by an inflammatory response, its diagnosis requires a high index of suspicion, a thorough evaluation of the patient’s medical history, and the exclusion of more common causes. Our patient’s recurrent presentation, absence of typical EBV symptoms, and normal findings on initial imaging underscore the importance of a comprehensive investigation, including serological and advanced imaging studies. This report emphasizes the need for early recognition and appropriate supportive care to prevent complications.
